# Sesquiterpene Lactams and Lactones With Antioxidant Potentials From *Atractylodes macrocephala* Discovered by Molecular Networking Strategy

**DOI:** 10.3389/fnut.2022.865257

**Published:** 2022-04-28

**Authors:** Pan Wang, Yi-nan Zhao, Rui-zhu Xu, Xiao-wei Zhang, Yi-ran Sun, Qing-mei Feng, Zhong-hua Li, Jiang-yan Xu, Zhi-shen Xie, Zhen-qiang Zhang, Heng-chao E

**Affiliations:** ^1^Academy of Chinese Medical Sciences, Henan University of Chinese Medicine, Zhengzhou, China; ^2^Henan Province Technological Innovation Center for Solid Preparation of Traditional Chinese Medicine, Zhongjing Wanxi Pharmaceutical Co., Ltd., Nanyang, China; ^3^College of Pharmacy, Henan University of Chinese Medicine, Zhengzhou, China; ^4^Institute for Agri-Food Standards and Testing Technology, Shanghai Academy of Agricultural Sciences, Shanghai, China

**Keywords:** sesquiterpene lactones, atractylenolactam, *Atractylodes macrocephala*, molecular networking, antioxidant activity

## Abstract

*Atractylodes macrocephala* rhizome (called Bái-zhú in China) has a long history as a functional food and herbal medicine in East Asia, especially China. Sesquiterpenoids are one of the main active compounds of *Atractylodes macrocephala* rhizome. This study aimed to explore the unknown sesquiterpenoids of *A. macrocephala* rhizome using a molecular networking strategy. Two new nitrogen-containing sesquiterpenoids, atractylenolactam A (**1**) and atractylenolactam B (**2**), and 2 new sesquiterpene lactones, 8-methoxy-atractylenolide V (**6**) and 15-acetoxyl atractylenolide III (**7**), along with 12 known analogs (**3-5** and **8-16**) were discovered and isolated. All the structures were assigned based on detailed spectroscopic analyses. The absolute configurations of **1**, **2**, **6**, and **7** were established by time-dependent density functional theory ECD (TDDFT-ECD) calculations. All these compounds had different degrees of concentration-dependent activating effects on nuclear-factor-E2-related factor-2 (Nrf2).

## Introduction

*Atractylodes macrocephala* (AM), a perennial herb, is distributed in many regions of China, Korea, and Japan (1). The rhizome of AM, named “*Báizhú*” in China, was first recorded in Shennong's Classic of Materia Medica (Shennong Bencao Jing, Dong Han Dynasty, A.D. 25–220). It is non-toxic and has been used for thousands of years to treat splenic disorders, dizziness, and heart palpitation (2). Nowadays, *Baizhu* is allowed to be developed and applied as a functional food by the National Health Commission, People's Republic of China (3). Scholarly research has shown that AM rhizome possesses a variety of biological activities, such as improving gastrointestinal (4), immunomodulatory (5), anti-Alzheimer's (6), anti-inflammatory (7), and anti-tumor activities (8). To date, about 230 members have been reported in AM rhizome, of which terpenoids and their glycosides are the main active ingredients (9).

Sesquiterpenes are the main components of terpenoids in AM rhizome, which are proven to have good antioxidant activity (10). In our subsequent study on active sesquiterpenoids, we isolated a variety of eudesmane sesquiterpenoids (11–13). Preliminary studies found many sesquiterpenoids from AM rhizome. How to quickly screen and replicate sesquiterpenoids is the key to the follow-up research on AM rhizome.

Mass spectrometry-based methods, which can accurately identify trace amounts of compounds in small amounts of material because of their high sensitivity and accuracy, have been widely used for the replication of natural products (14). The Global Natural Products Social Molecular Network (GNPS) is a global, interactive online platform and a mass spectrometry-based tool for natural products chemistry. Mass spectrometry data can be analyzed and grouped using GNPS, resulting in molecular networks and the annotation of the molecule (15). It has been proven effective by many studies, including sesquiterpenes discovery (16).

In this study, we applied a molecular network strategy to further explore the unknown sesquiterpenoids of AM rhizome. The application of this strategy led to the separation and identification of the following 16 sesquiterpenoids from AM rhizome: 2 new nitrogen-containing sesquiterpenoids, atractylenolactam A (No. **1**) and atractylenolactam B (No. **2**), 2 new sesquiterpene lactones 8-methoxy-atractylenolide V (No. **6**) and 15-acetoxyl atractylenolide III (No. **7**), and 12 known analogs atractylenolactam (No. **3**) (17), taenialactams A (No. **4**) (18), taenialactams B (No. **5**) (18), 15-epoxy-8β-hydroxyatractylenolide II (No. **8**) (19), atractylenolide I (No. **9**) (20–22), atractylenolide II (No. **10**) (21, 23, 24), 8-epiasterolid (No. **11**) (21, 25), atractylenolide III (No. **12**) (21, 26), 8-epiatractylenolide III (No. **13**) (19, 21), atractylenolide V (No. **14**) (27), 8β-ethoxyasterolid (No. **15**) (17, 23), and atractylenother (No. **16**) (19). Among them, nitrogen-containing sesquiterpenoids **4** and **5** were first isolated from plants. The high-resolution electrospray ionization mass spectrometry (HRESI-MS) and extensive spectroscopic data established these structures. The Nrf2 agonistic activity was investigated in HEK293T cells using the luciferase reporter assay.

## Materials and Methods

### General

The optical rotations were detected at 20°C using MCP 5100 digital polarimeter (Anton Paar, Graz, Austria). The UV data were recorded on a Shimadzu UV-2500 spectrophotometer (Shimadzu, Kyoto, Japan). The Chirascan CD spectrometer (Applied Photophysics Ltd., Surrey, UK) was used to acquire ECD spectra. 1D and 2D NMR spectra were recorded on a Bruker AVANCE III 500 M NMR spectrometer (Bruker BioSpin Corporation, Billerica, USA). UPLC-Q-TOF/MS analysis was carried out on a Waters ACQUITY UPLC system (Waters Corporation, Milford, USA) equipped with an AB SCIEX Triple TOF 5600 mass spectrometer with electrospray ionization source (ESI; Framingham, MA, USA). Silica gel (200–300 mesh, Qingdao Marine Chemical Inc., Qingdao, China), RP-C_18_ silica gel (Merck KGaA, Darmstadt, Germany), and Sephadex LH-20 gel (GE Healthcare Bio-Sciences AB, Uppsala, Sweden) were employed for column chromatography (CC). High-performance liquid chromatographies (HPLCs) were performed on Agilent 1260 series (Agilent Technologies, Santa Clara, USA) with C_18_ reversed-phase columns (YMC, Kyoto, Japan; 250 × 4.6 mm i.d., 5 μm, for analysis; 250 × 10 mm i.d., 5 μm, for separation).

### Plant Material

The dried rhizomes of AM were collected in Jiaozuo City, Henan Province. The sample was identified by Dr. Zhishen Xie. A voucher specimen (No. BZ1810) has been deposited at the Academy of Chinese Medical Sciences, Henan University of Chinese Medicine.

### UPLC-Q-TOF/MS Analysis

Sample separation was performed using an ACQUITY UPLC BEH C18 column (2.1 × 100 mm, 1.7 μm, Waters, Milford, MA, USA). The binary gradient elution system consisted of acetonitrile (A) and water containing 0.1% formic acid (B). Separation was achieved at the flow rate of 0.30 ml/min using the following program: initial, 5% A; 1 min, 5%A; 5 min, 60% A; 10 min, 70% A; 15 min, 95% A; 17 min, 95% A; 17.1 min 5% A; and 20 min 5% A. The sample injection volume was 3 μL and the column temperature was set at 35°C. All the samples were kept at 20°C during the analysis. Mass spectrometer analysis was used in positive ion modes. Data acquisition was performed in full scan mode (m/z ranges from 100 to 1,000) combined with IDA mode. The ion source was set with the following: ion source temperature, 500°C; spray voltage, 5,500 V; curtain gas pressure, 35 psi; gas 1 pressure, 50 psi; gas 2 pressure, 50 psi; the collision energy for TOF MS, 10 eV and for product ion, 40 eV; CES for product ion, 5 eV; ion release delay, 67; and ion release width, 25. The mass accuracy of the Q-TOF instrument during samples analysis was maintained by injecting APCI calibration solutions for each of the five samples. Auto IDA mode was utilized to acquire MS/MS data. The most intensive 8 ions from each TOF-MS scan were selected as AUTO MS/MS fragmentation precursors. Data acquisition was carried out using Analyst software (AB Sciex, v1.6).

### Molecular Network Analysis

The UPLC-Q-TOF-MS data were converted into mzXML files by using MSConvert (http://proteowizard.sourceforge.net) and then uploaded to the GNPS online platform to generate an MS/MS molecular network. Details of our molecular networking results are available at: https://gnps.ucsd.edu/ProteoSAFe/status.jsp?task=69301998b0a94ddfb66a64413b711158. Precursor-ion mass tolerance and fragment-ion tolerance were both set at 0.02 Da in this workflow. A molecular network was created with a cosine score above 0.7. Meanwhile, the matches were required for at least 6 matched fragment ions. Cytoscape 3.4.0 was used for network visualization.

### Extraction and Isolation

The dried rhizomes of AM (2 kg) were extracted with 95% ethanol at room temperature (3 × 5 L, 7 d for each time) to afford the ethanol extract, which was then partitioned between water and ethyl acetate (5 × 2 L) to afford the ethyl acetate soluble extract (150 g). The ethyl acetate extract was subjected to silica gel column chromatography (150 × 12.0 cm i.d.) with a gradient mixture of CH_2_Cl_2_-CH_3_OH (100:0–5:1) to give 10 fractions (Fr. 1–Fr. 10). Fr. 4 (5.6 g) was purified by silica gel eluted with gradient petroleum ether–ethyl acetate (50:1–1:1) to give eight fractions (Fr. 4.1–Fr. 4.8). Fr. 4.2 (100.0 mg) and Fr. 4.3 (1.0 g) recrystallized to yield compound **16** (10.0 mg) and compound **9** (500.0 mg), respectively. Fr. 4.4 (2.5 g) was further subjected to a C_18_ reversed-phase column (CH_3_OH-H_2_O, 1:1–1:0) and purified by preparative HPLC (CH_3_OH-H_2_O 75:25, 3 mL/min) to yield compound **10** (12.1 mg, t_R_ = 5.8 min), compound **15** (16.0 mg, t_R_ = 15.6 min), and compound **11** (3.0 mg, t_R_ = 15.9 min). Fr. 4.6 (1.5 g) was subjected to a C_18_ reversed-phase column (CH_3_OH-H_2_O, 1:1–1:0) and further purified by preparative HPLC (CH_3_OH-H_2_O 70:30, 3 mL/min) to yield compound **14** (180.0 mg, t_R_ = 4.2 min), compound **8** (5.0 mg, t_R_ = 6.2 min), compound **7** (3.0 mg, t_R_ = 8.9 min), compound **12** (180.0 mg, t_R_ = 10.2 min), and compound **13** (2.0 mg, t_R_ = 10.8 min). Fr. 6 (32.8 g) was separated on an MCI reversed-phase column (CH_3_OH-H_2_O, 1:1 to 1:0) to afford five fractions (Fr. 6.1–Fr. 6.5). Fr. 6.3 (5.0 g) was separated by silica gel eluted with gradient petroleum ether–ethyl acetate (5:1–1:4) to yield Fr. 6.3.1–Fr. 6.3.8. Fr. 6.3.1 (50.0 mg) were purified by preparative HPLC (CNCH_3_-H_2_O 50:50, 3 mL/min) to yield compound **6** (5.3 mg, 24.2 min). Fr. 6.3.3 (250.0 mg) was separated on a preparative HPLC (CH_3_OH-H_2_O 65:35, 3 mL/min) to yield compound **2** (3.1 mg, t_R_ = 10.2 min), compound **5** (5.3 mg, t_R_ = 14.2 min), compound **1** (5.0 mg, t_R_ = 20.9 min), compound **4** (2.9 mg, t_R_ = 21.6 min), and compound **3** (10.0 mg, t_R_ = 24.2 min). All these compounds are listed in **Figure 2**.

### Structure Identification

Compound **1**: White amorphous powder; [α]_20 D_ + 78.0 (*c* 0.1, MeOH); UV (MeOH) λ_max_ (log ε) 275 (3.51) nm; ECD (*c* 0.35 mM, MeOH) λ_max_ (Δε) 203 (– 0.27), 268 (+ 0.59) nm; ^1^H NMR data see Table 1 and ^13^C NMR data see Table 2; HRESIMS *m/z* 288.1586 [M + H]^+^ (calcd for C_17_H_22_NO_3_, 288.1594).

**Table 1:** ^1^H NMR (500 MHz) data for new compounds **1**, **2**, **6**, and **7** in CDCl_3_ solvent (δ in ppm, *J* in Hz)^*a*^.

**No**	**1 (δ)**	**2(δ)**	**6(δ)**	**7(δ)**
1-β	1.71 m	2.03 m	2.05 td (*J* = 13.0, 5.0)	1.54 m
1-α	1.71 m	1.47 br d (*J* = 12.7)	1.30 br d (*J* = 13.0)	1.35 m
2-β	2.03 m	1.68 m	1.66 m	2.17 m
2-α	1.69 m	1.68 m	1.66 m	2.17 m
3	5.17 m	β 2.33 br t (*J* = 12.5) α 1.97 m	β 2.34 br d (*J* = 13.0) α 1.96 td (*J* = 13.0, 6.0)	5.82 br s
5	2.31 dd (*J* = 13.0, 3.5)	2.30 ove	2.32 ove	2.34 ove
6-β	2.52 dd (*J* = 16.5, 13.0)	2.17 br t (*J* = 13.0)	2.25 br d (*J* = 12.5)	2.31 br d (13.0)
6-α	2.65 dd (*J* = 16.5, 3.5)	2.55 dd (*J* = 13.0, 3.0)	2.60 dd (*J* = 12.5, 3.0)	2.81 dd (*J* = 13.0, 3.0)
9	5.45 s	3.54 s	3.70 s	β 2.26 d (*J* = 13.5) α 1.52 d (*J* = 13.5)
13	1.88 s	1.86 s	1.90 s	1.83 s
14	0.93 s	1.02 s	1.00 s	1.13 s
15-*a*	5.05 s	4.87 s	4.88 s	4.61 d (*J* = 12.0)
15-*b*	4.79 s	4.61 s	4.59 s	4.51 d (*J* = 12.0)
1′	2.15 s			2.08 s
1″			3.21 s	
1^‴^		3.11 s		

a *Overlapping signals assigned by ^1^H–^1^H COSY, HSQC, and HMBC spectra without designating multiplicity*.

**Table 2:** ^13^C NMR (125 MHz) data for new compounds **1**, **2**, **6**, and **7** in CDCl_3_ solvent (δ in ppm).

**No**	**1(δ)**	**2(δ)**	**6(δ)**	**7(δ)**
1	37.0, CH_2_	34.8, CH_2_	34.6, CH_2_	36.8, CH_2_
2	29.0, CH_2_	22.2, CH_2_	22.1, CH_2_	22.7, CH_2_
3	73.8, CH	36.1, CH_2_	36.1, CH_2_	129.4, CH
4	145.9, C	149.7, C	149.1, C	131.5, C
5	47.2, CH	44.0, CH	44.4, CH	46.6, CH
6	22.2, CH_2_	24.3, CH_2_	24.8, CH_2_	23.6, CH_2_
7	140.7, C	151.9, C	151.5, C	160.5, C
8	135.6, C	93.2, C	107.9, C	103.4, C
9	119.1, CH	78.7, CH	78.0, CH	50.6, CH_2_
10	37.6, C	40.6, C	40.7, C	33.3, C
11	125.4, C	130.0, C	126.2, C	122.6, C
12	172.9, C	174.5, C	172.0, C	171.8, C
13	8.2, CH_3_	8.0, CH_3_	8.4, CH_3_	8.2, CH_3_
14	18.5, CH_3_	16.2, CH_3_	15.9, CH_3_	15.8, CH_3_
15	105.0, CH_2_	106.6, CH_2_	106.9, CH_2_	67.0, CH_2_
1'	170.0, C			170.7, C
2'	21.1, CH_3_			21.0, CH_3_
1”			50.4, CH_3_	
1”'		49.6, CH_3_		

Compound **2:** White amorphous powder; [α]_20 D_ + 100.0 (*c* 0.1, MeOH); UV (MeOH) λ_max_ (log ε) 205 (3.62) nm; ECD (*c* 0.36 mM, MeOH) λ_max_ (Δε) 196 (– 3.36), 220 (+ 2.74), 253 (+ 1.22) nm; ^1^H NMR data see Table 1 and ^13^C NMR data see Table 2; HRESIMS *m/z* 278.1745 [M + H]^+^ (calcd for C_16_H_24_NO_3_, 278.1751).

Compound **6:** White amorphous powder; [α]_20 D_ + 201.0 (*c* 0.1, MeOH); UV (MeOH) λ_max_ (log ε) 221 (3.29) nm; ECD (*c* 0.36 mM, MeOH) λ_max_ (Δε) 210 (+ 1.62), 220 (+ 1.25), 242 (+ 3.42) nm; ^1^H NMR data see Table 1 and ^13^C NMR data see Table 2; HRESIMS *m/z* 279.1588 [M + H]^+^ (calcd for C_16_H_23_O_4_, 279.1591).

Compound **7:** White amorphous powder; [α]_20 D_ – 43.0 (*c* 0.1, MeOH); UV (MeOH) λ_max_ (log ε) 204 (3.26) nm; ECD (*c* 0.33 mM, MeOH) λ_max_ (Δε) 215 (+ 0.52), 223 (+ 0.40), 243 (+ 1.64) nm; ^1^H NMR data see Table 1 and ^13^C NMR data see Table 2; HRESIMS *m/z* 307.1532 [M + H]^+^ (calcd for C_17_H_23_O_5_, 307.1540).

### Luciferase Assays

The assay for Nrf2 agonistic activities was achieved by luciferase-reported gene methods (28). An expression vector containing the ARE-promoter region of the reporter gene firefly luciferase pGL4 (luc2P/NRF2/Hygro) was transfected into HEK293T cells momentarily. Transfections were performed according to the instructions using Lipofectamine 2000 transfection reagent (Invitrogen, Chicago, IL, USA). At 24 h post-transfection, the transfected cells were treated with compounds (10–30 μM) for 18 h and assayed using the luciferase reporter assay kit (Promega, US).

## Results and Discussion

### Sesquiterpenoids Discovery in the Rhizome of AM

**Figure 1:**
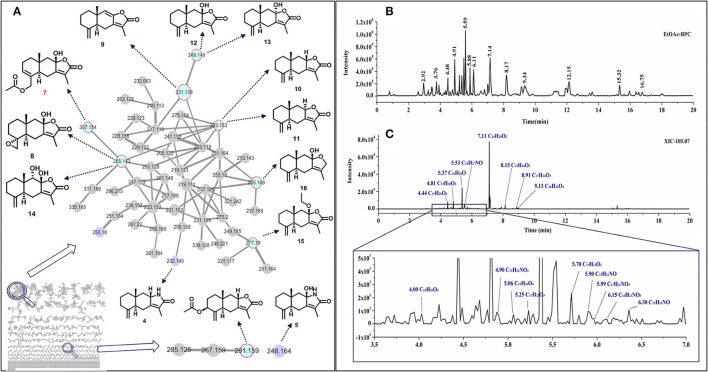
Sesquiterpenoids discovery in the rhizome of AM by GNPS and metabolic profiling analysis. GNPS analysis of AM methanol extract ethyl acetate extract layer. Blue nodes represented compounds that have been identified, purple nodes represented nitrogen-containing sesquiterpenoids, gray nodes remained unknown **(A)**, BPC of UPLC-QTOF-MS/MS spectra **(B)**, XIC of 105.07 with product-ion spectra **(C)**.

Sesquiterpenoids are the main components in the rhizome of AM. We performed a GNPS study to search and dereplicate sesquiterpenoids in the rhizome of AM. After removing 1-node-clusters, 445 clusters were produced using the data obtained from UPLC-Q-TOF-MS/MS with the AM extracts (Figure 1A). Based on the library search results, cluster 1 (Figure 1A) and cluster 110 (Figure 1A) were identified as containing sesquiterpenes. In these 2 clusters, we matched the chemical formula of 49 nodes (Supplementary Table 1). Interestingly, 3 N-containing sesquiterpenoids or derivatives were found in AM were present in the results, and they were uncommonly discovered in AM. Further analyzing these 49 nodes with MS/MS spectra, we found that a fragment of m/z 105.07 was contained in almost all nodes. This fragment was likely to be the characteristic fragment ion in the mass spectrometry detection of sesquiterpenoids in AM. Therefore, an BPC of rhizome of AM (Figure 1B) and XIC of m/z 105.07 was performed with MS/MS spectra (Figure 1C). Another 6 sesquiterpenoids were found, including 5 N-containing sesquiterpenes. Since sesquiterpenoids with the same molecular formula had isomers, further analysis such as purification and identification need to be performed with these compounds.

### Structure Identification

**Figure 2:**
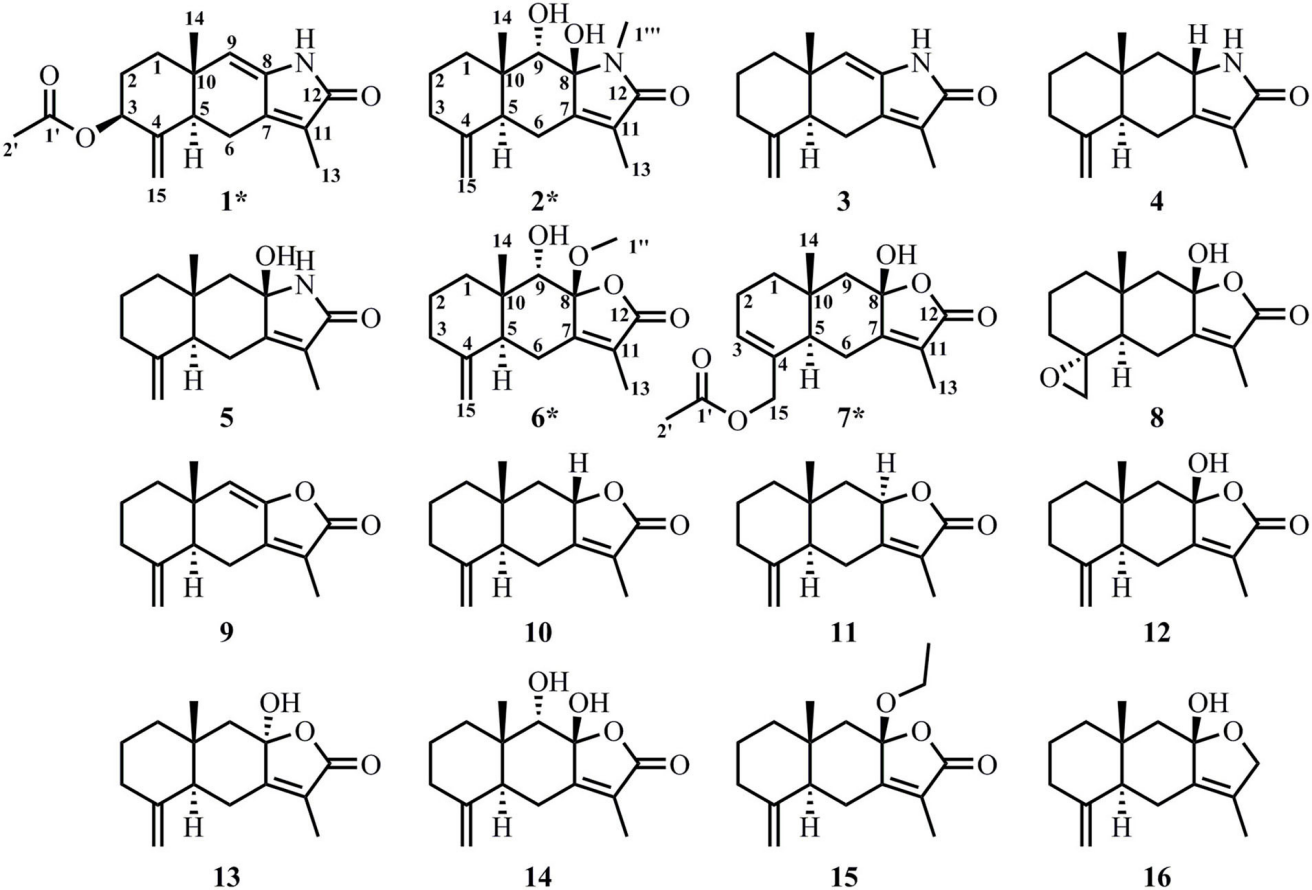
Structures of compounds **1**–**16** identified in the rhizome of AM. The new compounds are marked with *.

Guided by metabolic profiling of sesquiterpenoids in rhizome of AM, we identified 16 compounds in this study (Figure 2).

**Figure 3:**
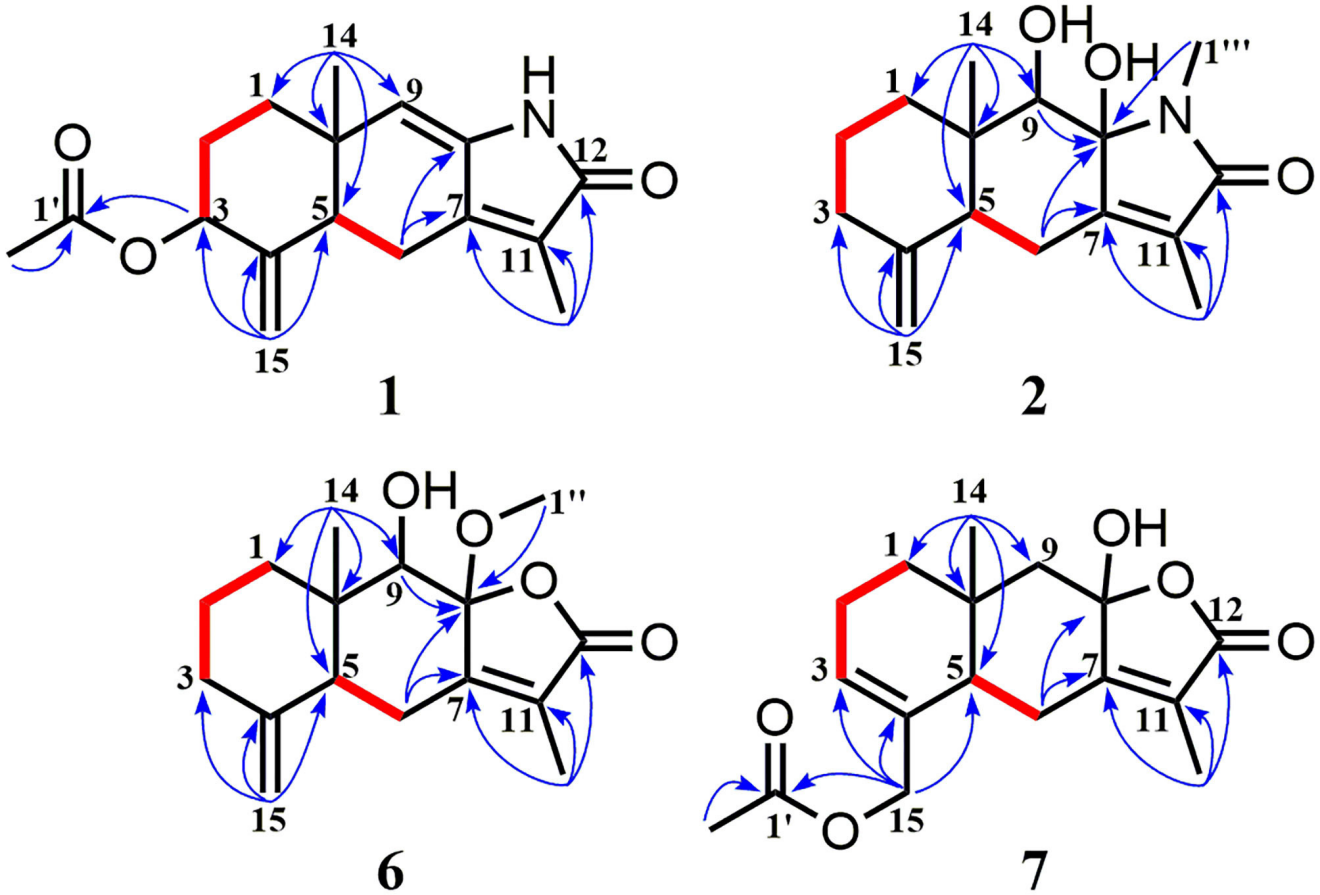
^1^H-^1^H COSY (bold red bonds) and Key HMBC (blue arrows) correlations for new compounds **1**, **2**, **6**, and **7**.

**Figure 4:**
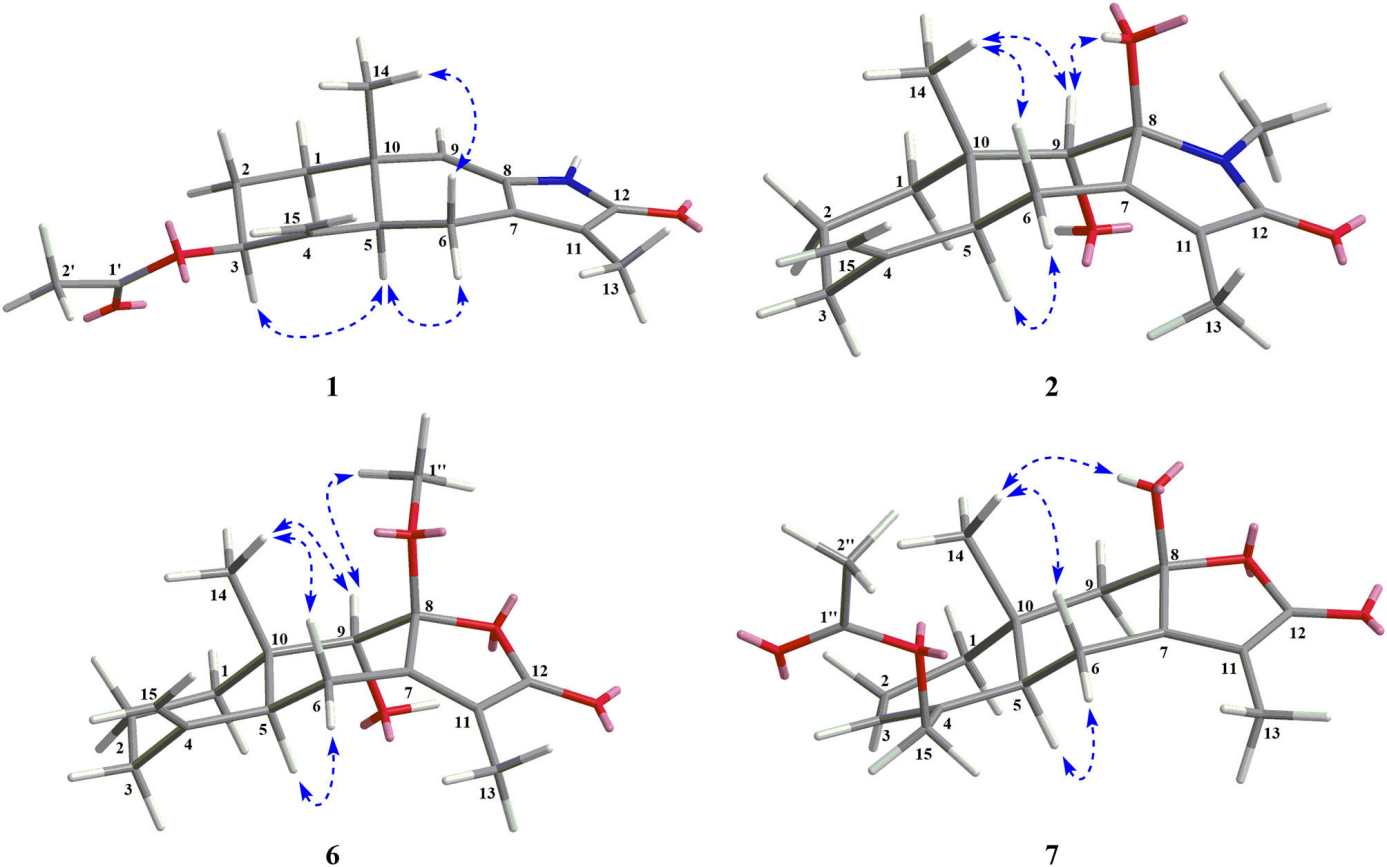
Key NOE interactions for new compounds **1**, **2**, **6**, and **7**.

**Figure 5:**
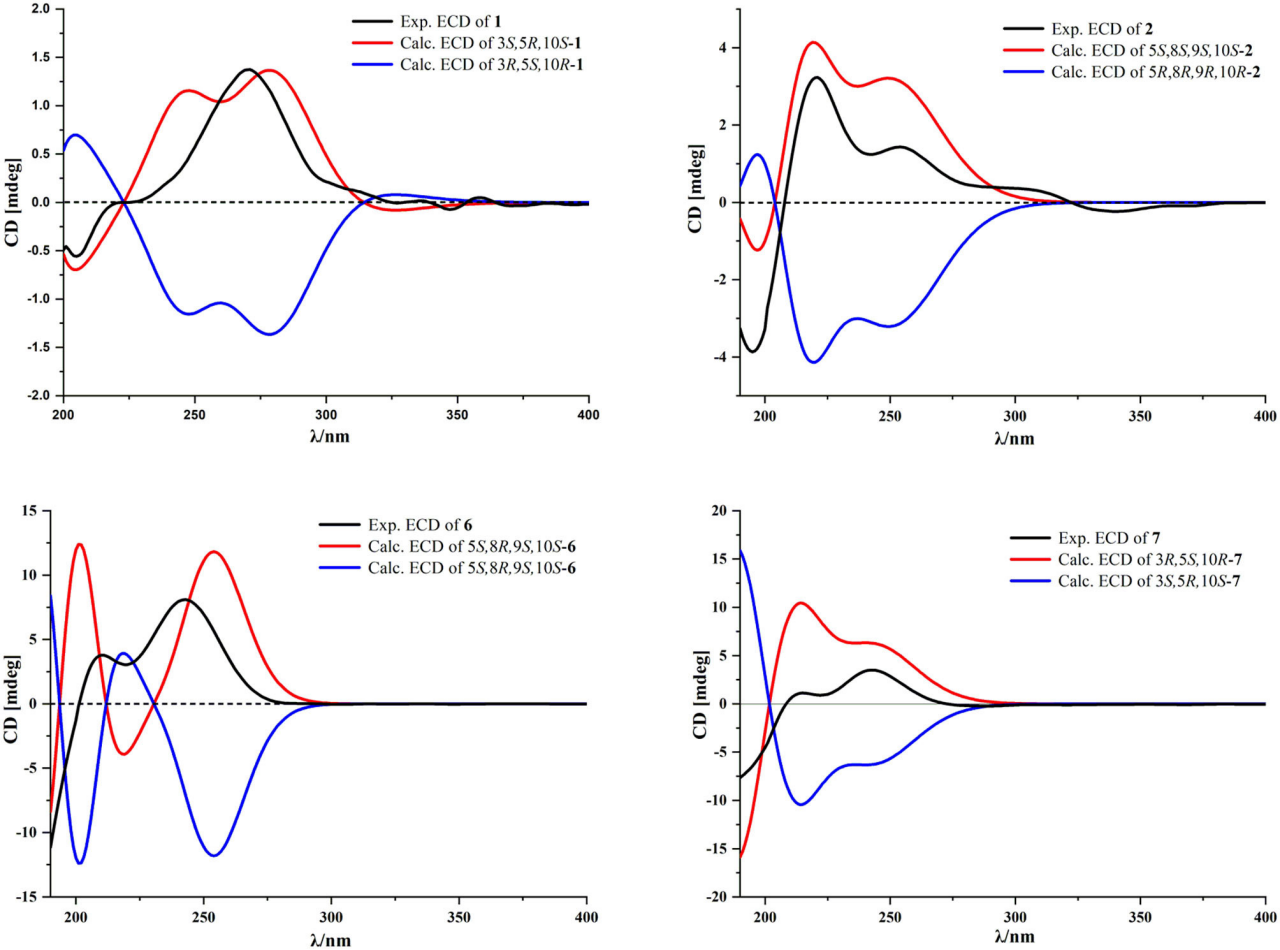
Experimental and calculated ECD spectra of new compounds **1**, **2**, **6**, and **7**.

Compound **1** was isolated as a white amorphous powder. Its molecular formula was assigned as C_17_H_21_NO_3_ on the basis of the positive HRESIMS ion at *m/z* 288.1586 ([M + H]^+^, calcd for C_17_H_22_NO_3_, 288.1594), indicating eight degrees of unsaturation. In the ^1^H NMR (Table 1), signals of an acetylmethyl group [δ_H_ 2.15 (3H, s)], two methyl protons [δ_H_ 1.88 (3H, s), 0.93 (3H, s)], four methylene groups [δ_H_ 5.05 (1H, s), 4.79 (1H, s), 2.65 (1H, dd, *J* = 16.5, 3.5 Hz), 2.52 (1H, dd, *J* = 16.5, 13.0 Hz), 2.03 (1H, m), 1.71 (2H, m), 1.69 (1H, m)], and three methine protons [δ_H_ 5.45 (1H, s), 5.17 (1H, m), 2.31 (1H, dd, *J* = 13.0, 3.5 Hz)], were observed. The ^13^C NMR (Table 2) and HSQC data discovered 16 resonances corresponding to an acetylmethyl group (δ_C_ 21.1), two methyl groups (δ_C_ 18.5, 8.2), four methylene moieties (δ_C_ 105.0, 37.0, 29.0, 22.2), three methine groups (δ_C_ 119.1, 73.8, 47.2), and seven quaternary carbons (δ_C_ 172.9, 170.0, 145.9, 140.7, 135.6, 125.4, 37.6). The NMR data of **1** were very similar to those of atractylenolactam (14), except for the presence of an acetyl group [δ_H_ 2.15 (3H, s), δ_C_ 170.0, 21.1] and a hypoxy methylene [δ_H_ 5.17 (1H, m), δ_C_ 73.8] in **1**, and the absence of a methylene [δ_H_ 2.37 (1H, dd, *J* = 14.7, 3.3 Hz), 2.05 (1H, m), δ_C_ 36.3] in atractylenolactam, which indicated an acetylation atractylenolactam. Analyses of the 2D ^1^H-^1^H COSY and HMBC NMR data (Figure 3) confirmed the planar structure of **1** as shown. ^1^H-^1^H COSY correlations between H-3/H_2_-2 and pivotal HMBC correlation from H-3 to C-1' assigned the acetyl group as CH-3. The relative configuration of **1** was assigned by the key NOE interaction (Figure 4), and the NOE cross-peaks between H-6b/H_3_-14 are supportive of their cofacial relationship and the arbitrarily assigned as β-orientation, whereas those between H-6a/H-5 and H-6a/H-3 revealed the α-orientation. The absolute configuration of **1** was determined by comparing its experimental ECD spectrum with the calculated one predicted by TDDFT method. The experimental and calculated ECD spectra (Figure 5) of **1** were in good agreement. Therefore, the absolute configuration of **1** was identified as 3*S*,5*R*,10*S*.

Compound **2** was obtained as a white amorphous powder. The molecular formula of **2** was determined to be C_16_H_23_NO_3_ by the quasi-molecular ion at *m/z* 278.1745 [M + H]^+^ in its HRESIMS (calcd for C_16_H_24_NO_3_, 278.1751), implying that it has six degrees of unsaturation. The NMR data (Tables 1, 2) of **2** were very similar to its co-metabolite, the taenialactams B (**5**) (15), except for the presence of an oxygenated methine moiety [δ_H_ 3.54 (1H, s), δ_C_ 78.7] and a nitrogenous methylene group [δ_H_ 3.11 (3H, s), δ_C_ 49.6]. HMBC correlations (Figure 3) from H_3_-14 to C-1/C-5/C-9/C-10 and H_3_-1”'/H-9 to C-8 revealed hydroxyl group at the C-9 and the methyl group at nitrogen atom. The NOESY cross peaks between H_3_-14/H-6b, H_3_-14/H-9, and 8-OH/H-9 indicated these protons were cofacial (Figure 4). The 5*S*,8*S*,9*S*,10*S* configuration of **2** was defined based on the agreement between the experimental and calculated ECD curves (Figure 5).

Compound **6** exhibited a molecular formula of C_16_H_22_O_4_ as deduced from its positive HRESIMS ion at *m/z* 279.1588 [M + H]^+^ (calcd for C_16_H_23_O_4_, 279.1591). Its NMR data (Tables 1, 2) were similar to those of atractylenolide V (24). The main difference was the replacement of a hydroxyl group in atractylenolide V by a methoxyl group in **6** (δ_H_ 3.21 s, δ_C_ 50.4), whose presence was further supported by key HMBC correlations (Figure 3) from H_3_-1” (δ_H_ 3.21) to C-8. The absolute configuration of **6** was established as 5*S*,8*R*,9*S*,10*S* by comparing its experimental ECD spectrum (Figure 5) with the calculated one.

Compound **7** was found to be isomers with the same molecular formula of C_17_H_22_O_5_, based on a combination of ^13^C NMR and HRESIMS data *m/z* 307.1532 [M + H]^+^ (calcd for C_17_H_23_O_5_, 307.1540). Analysis of the NMR data (Tables 1, 2) of **7** and comparison with those of atractylenolide III (18, 23) revealed that the extra-ring double bond in atractylenolide III was rearranged and oxidized to acetyl groups, which was substantiated by its HMBC correlations (Figure 3) from H_2_-15 (δ_H_ 4.61, 4.51) to C-1'/C-3/C-4. The relative configuration of **7** was determined by the explanation of their NOESY data (Figure 4). NOESY correlations between H-5/H-6a, H-6b/H_3_-14, and H_3_-14/8-OH indicated that H_3_-14 and 8-OH were cofacial. The absolute configuration of **7** was determined to be 3*R*,5*S*,10*R* by comparing their experimental and calculated ECD spectra (Figure 5).

The structures of known compounds **3**–**5** and **8**–**16** were established by comparing their NMR data to previously reported data.

### Anti-oxidative Activities

**Figure 6:**
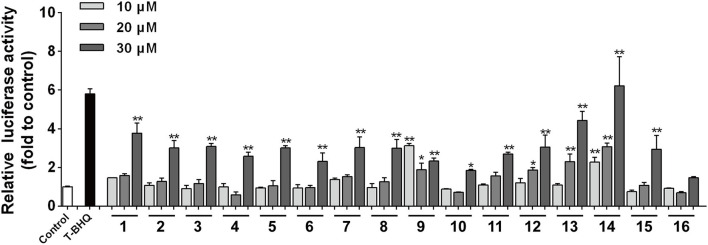
The agonistic activities of compounds **1**–**16** on Nrf2 (*n* = 3). All compounds showed concentration-dependent antioxidant activity. *p-value < 0.05 and **p-value < 0.01.

The activation of the Nrf2 signaling pathway plays an important role in the antioxidant process (29). The antioxidant activity is closely related with the traditional use of *Baizhu* for improving longevity (30). The Nrf2 activation activity of AM rhizome was therefore chosen for exploration in this study. All the isolates were evaluated for their Nrf2-activating (Figure 6) effects with T-BHQ as the positive control at a dosing concentration of 10 μM. All these compounds had different degrees of dosing-dependent activation effect on Nrf2. However, no significant difference was found in the activity comparison results between the 16 compounds, indicating that the antioxidant effects of these compounds were universal. These sesquiterpenes, the main components of non-toxic Atractylodes rhizoma, had the potential to be developed into functional food ingredients.

## Conclusion

In our subsequent study on active sesquiterpenoids from the natural world, we had isolated a variety of eudesmane sesquiterpenoids from AM rhizome. The MS/MS spectra of these sesquiterpenoids presented the same cleavage fragments. In this study, we presented a molecular network strategy to further explore the unknown eudesmane sesquiterpenoids of AM rhizome. The application of this strategy led to the discovery of 5 nitrogen-containing sesquiterpenoids including 2 new ones (**1** and **2**), 2 analogs isolated from plants for the first time (**4** and **5**), and 11 sesquiterpene lactones counting 2 new ones (**6** and **7**). This strategy can greatly improve the efficiency of identifying sesquiterpenes in the AM rhizome and can also be used for the discovery of sesquiterpenoids in other functional foods. All compound structures in this study were assigned on the basis of detailed spectroscopic analyses. The absolute configuration of new compounds was established by comparing the experimental and the calculated ECD spectra. The Nrf2 agonistic activity was investigated in HEK293T cells using the dual-luciferase reporter assay. The bioactivity study showed that all compounds exhibited concentration-dependent activation effects on Nrf2, implying that all 16 sesquiterpenoids had an antioxidant activity. This study has provided a scientific basis for the use of AM as a possible functional food.

## Data Availability Statement

The original contributions presented in the study are included in the article/Supplementary Material, further inquiries can be directed to the corresponding authors.

## Author Contributions

H-cE, Z-qZ, and Z-sX designed the research. PW and Y-nZ responsible for data analysis and article writing. R-zX conducted chemical experiments. X-wZ and Y-rS responsible for biology experiments. Q-mF and Z-hL contributes to ECD experiments. J-yX is responsible for the theoretical guidance of Chinese medicine. All authors contributed to the article and approved the submitted version.

## Funding

This work was financially supported by grants from the Project funded by China Postdoctoral Science Foundation (No. 2021M693704), National Natural Science Foundation of China (No. 82004019), Scientific and Technological Project of Science and Technology Department of Henan Province (Nos. 202102310183 and 202102310173), Central Plains Thousand Talents Plan Leading Talents of Science and Technology Innovation in Henan Province (No. 204200510022), Program for Innovative Research Team in University of Henan Province (No. 21IRTSTHN026) and the Special Research Project of Henan Province on Traditional Chinese Medicine (No. 2018ZYZD12).

## Conflict of Interest

PW was employed by Zhongjing Wanxi Pharmaceutical Co., Ltd. The remaining authors declare that the research was conducted in the absence of any commercial or financial relationships that could be construed as a potential conflict of interest.

## Publisher's Note

All claims expressed in this article are solely those of the authors and do not necessarily represent those of their affiliated organizations, or those of the publisher, the editors and the reviewers. Any product that may be evaluated in this article, or claim that may be made by its manufacturer, is not guaranteed or endorsed by the publisher.
