# Supplementary material for: Sesquiterpene Lactams and Lactones With Antioxidant Potentials From Atractylodes macrocephala Discovered by Molecular Networking Strategy
Source: Front Nutr. 2022 Apr 28;9:865257. doi: 10.3389/fnut.2022.865257 (PMC9097160; doi:10.3389/fnut.2022.865257)
Supplement: Supplementary file 1 [file Data_Sheet_1.docx]

Supplementary Material

Sesquiterpene Lactams and Lactones with Antioxidant Potentials from *Atractylodes Macrocephala* Discovered by Molecular Networking Strategy

Pan Wang ^1,2†^, Yi-nan Zhao^3†^, Rui-zhu Xu^3^, Xiao-wei Zhang^1^, Yi-ran Sun^1^, Qing-mei Feng^1^, Zhong-hua Li^1^, Jiang-yan Xu^1^, Zhi-shen Xie^1*^, Zhen-qiang Zhang^1*^ and Heng-chao E^4*^

^1^ Academy of Chinese Medical Sciences, Henan University of Chinese Medicine, Zhengzhou 450046, P. R. China

^2^ Henan Province Technological Innovation Center for Solid Preparation of Traditional Chinese Medicine, Zhongjing Wanxi Pharmaceutical Co., Ltd., Nanyang 474500, P.R. China

^3^ College of Pharmacy, Henan University of Chinese Medicine, Zhengzhou 450046, P. R. China

^4^ Institute for Agri-Food Standards and Testing Technology, Shanghai Academy of Agricultural Sciences, Shanghai, 201403, P. R. China

# ^†^ These authors have contributed equally to this work and share first authorship.

*** Correspondence:**Zhi-shen xie
xiezhishen_1988@163.com

Zhen-qiang Zhang
[zhang_zhenqiang@126.com](mailto:zhang_zhenqiang@126.com)

Heng-chao E
ehengchao@126.com

|  | **Table of contents** | 1 |
| --- | --- | --- |
| **Table S1** | MS details of nodes in partial cluster 1 and cluster 110 | 2-3 |
| **Figure S1** | UV spectrum of compound **1** | 4 |
| **Figure S2** | HRESIMS spectrum of compound **1** | 4 |
| **Figure S3** | ^1^H NMR (500 MHz) spectrum of compound **1** in CDCl_3_ | 5 |
| **Figure S4** | ^13^C NMR (125 MHz) spectrum of compound **1** in CDCl_3_ | 5 |
| **Figure S5** | DEPT135 (125 MHz) spectrum of compound **1** in CDCl_3_ | 6 |
| **Figure S6** | ^1^H-^1^H COSY (500 MHz) spectrum of compound **1** in CDCl_3_ | 6 |
| **Figure S7** | HSQC (500 MHz) spectrum of compound **1** in CDCl_3_ | 7 |
| **Figure S8** | HMBC (500 MHz) spectrum of compound **1** in CDCl_3_ | 7 |
| **Figure S9** | NOESY (500 MHz) spectrum of compound **1** in CDCl_3_ | 8 |
| **Figure S10** | UV spectrum of compound **2** | 9 |
| **Figure S11** | HRESIMS spectrum of compound **2** | 9 |
| **Figure S12** | ^1^H NMR (500 MHz) spectrum of compound **2** in CDCl_3_ | 10 |
| **Figure S13** | ^13^C NMR (125 MHz) spectrum of compound **2** in CDCl_3_ | 10 |
| **Figure S14** | DEPT135 (125 MHz) spectrum of compound **2** in CDCl_3_ | 11 |
| **Figure S15** | ^1^H-^1^H COSY (500 MHz) spectrum of compound **2** in CDCl_3_ | 11 |
| **Figure S16** | HSQC (500 MHz) spectrum of compound **2** in CDCl_3_ | 12 |
| **Figure S17** | HMBC (500 MHz) spectrum of compound **2** in CDCl_3_ | 12 |
| **Figure S18** | NOESY (500 MHz) spectrum of compound **2** in CDCl_3_ | 13 |
| **Figure S19** | ^1^H NMR (500 MHz) spectrum of compound **2** in DMSO-*d*_6_ | 13 |
| **Figure S20** | ^13^C NMR (125 MHz) spectrum of compound **2** in DMSO-*d*_6_ | 14 |
| **Figure S21** | DEPT135 (125 MHz) spectrum of compound **2** in DMSO-*d*_6_ | 14 |
| **Figure S22** | ^1^H-^1^H COSY (500 MHz) spectrum of compound **2** in DMSO-*d*_6_ | 15 |
| **Figure S23** | HSQC (500 MHz) spectrum of compound **2** in DMSO-*d*_6_ | 15 |
| **Figure S24** | HMBC (500 MHz) spectrum of compound **2** in DMSO-*d*_6_ | 16 |
| **Figure S25** | ROESY (500 MHz) spectrum of compound **2** in DMSO-*d*_6_ | 16 |
| **Figure S26** | UV spectrum of compound **6** | 17 |
| **Figure S27** | HRESIMS spectrum of compound **6** | 17 |
| **Figure S28** | ^1^H NMR (500 MHz) spectrum of compound **6** in CDCl_3_ | 18 |
| **Figure S29** | ^13^C NMR (125 MHz) spectrum of compound **6** in CDCl_3_ | 18 |
| **Figure S30** | DEPT135 (125 MHz) spectrum of compound **6** in CDCl_3_ | 19 |
| **Figure S31** | ^1^H-^1^H COSY (500 MHz) spectrum of compound **6** in CDCl_3_ | 19 |
| **Figure S32** | HSQC (500 MHz) spectrum of compound **6** in CDCl_3_ | 20 |
| **Figure S33** | HMBC (500 MHz) spectrum of compound **6** in CDCl_3_ | 20 |
| **Figure S34** | NOESY (500 MHz) spectrum of compound **6** in CDCl_3_ | 21 |
| **Figure S35** | UV spectrum of compound **7** | 22 |
| **Figure S36** | HRESIMS spectrum of compound **7** | 22 |
| **Figure S37** | ^1^H NMR (500 MHz) spectrum of compound **7** in CDCl_3_ | 23 |
| **Figure S38** | ^13^C NMR (125 MHz) spectrum of compound **7** in CDCl_3_ | 23 |
| **Figure S39** | DEPT135 (125 MHz) spectrum of compound **7** in CDCl_3_ | 24 |
| **Figure S40** | ^1^H-^1^H COSY (500 MHz) spectrum of compound **7** in CDCl_3_ | 24 |
| **Figure S41** | HSQC (500 MHz) spectrum of compound **7** in CDCl_3_ | 25 |
| **Figure S42** | HMBC (500 MHz) spectrum of compound **7** in CDCl_3_ | 25 |
| **Figure S43** | NOESY (500 MHz) spectrum of compound **7** in CDCl_3_ | 26 |
| **Figure S44** | ^1^H NMR (500 MHz) spectrum of compound **7** in DMSO-*d*_6_ | 26 |
| **Figure S45** | ROESY (500 MHz) spectrum of compound **7** in DMSO-*d*_6_ | 27 |

**Table S1 MS details of nodes in partial cluster 1 and cluster 110.**

| **No.** | **Node** | **Formula** | **Exact mass** | **[M+H]^+^** | **MS/MS product ion** |
| --- | --- | --- | --- | --- | --- |
| 1 | 205.122 | C13H16O2 | 204.1150 | 205.1223 | 105.07, 115.05, 129.07, 131.08, 159.11, 187.11 |
| 2 | 205.158 | C14H20O | 204.1514 | 205.1587 | 105.07, 145.10, 159.11, 177.14, 187.14 |
| 3 | 217.118 | C14H16O2 | 216.1150 | 217.1223 | 105.07, 115.05, 128.06, 129.07, 141.07, 199.12 |
| 4 | 219.133 | C14H18O2 | 218.1307 | 219.1380 | 105.07, 119.08, 129.07, 131.08, 145.09, 201.14 |
| 5 | 219.174 | C15H22O | 218.1671 | 219.1743 | 105.07, 119.08, 131.08, 145.10, 177.13, 201.15 |
| 6 | 221.117 | C13H16O3 | 220.1099 | 221.1172 | 105.07, 119.08, 133.10, 142.08 |
| 7 | 221.153 | C14H20O2 | 220.1463 | 221.1536 | 105.07, 119.08, 161.12, 178.08, 203.13 |
| 8 | 221.189 | C15H24O | 220.1827 | 221.1900 | 105.07, 119.08, 133.10, 178.08, 203.17 |
| 9 | 229.122 | C15H16O2 | 228.1150 | 229.1223 | 115.05, 128.06, 129.07, 131.08, 153.07, 199.10 |
| 10 | 229.158 | C16H20O | 228.1514 | 229.1587 | 115.05, 128.06, 129.07, 141.07, 153.07, 199.10, 201,12 |
| 11 | 231.139 | C15H18O2 | 230.1307 | 231.1380 | 105.07, 128.06, 129.07, 142.08, 143.08, 185.13, 213.13 |
| 12 | 232.145 | C15H21NO | 231.1623 | 232.1696 | 105.07, 119.09, 133.06, 189.12, 199.11, 214.12 |
| 13 | 233.063 | C13H12O4 | 232.0736 | 233.0808 | 105.07, 115.05, 128.06, 129.07, 145.09, 203.06 |
| 14 | 233.118 | C14H16O3 | 232.1099 | 233.1172 | 105.07, 128.06, 129.07, 131.08, 145.09, 187.11, 215.11 |
| 15 | 233.153a | C15H20O2 | 232.1463 | 233.1536 | 105.07, 131.08, 145.10, 159.11, 215.12 |
| 16 | 233.153b | C15H20O2 | 232.1463 | 233.1536 | 105.07, 119.08, 128.06, 131.07, 145.09, 159.09, 215.15, 274.83 |
| 17 | 235.168 | C15H22O2 | 234.1620 | 235.1693 | 105.07, 119.08, 131.08, 159.11, 205.12, 217.13 |
| 18 | 237.185 | C15H24O2 | 236.1776 | 237.1849 | 105.07, 119.08, 145.10, 159.11, 177.11, 191.12, 201.15, 219.15 |
| 19 | 239.128 | C13H18O4 | 238.1205 | 239.1278 | 105.07, 119.08, 133.09, 179.10 |
| 20 | 247.132 | C15H18O3 | 246.1256 | 247.1329 | 105.07, 128.06, 129.07, 131.09, 145.10, 201.12, 219.12, 229.12 |
| 21 | 247.173 | C16H22O2 | 246.1620 | 247.1693 | 125.06, 131.09, 145.10, 159.11, 173.11, 187.14, 229.15 |
| 22 | 248.164 | C15H21NO2 | 247.1572 | 248.1645 | 128.06, 143.08, 157.10, 231.14 |
| 23 | 249.113 | C14H16O4 | 248.1049 | 249.1121 | 105.07, 115.05, 128.06, 129.07, 141.07, 189.09, 203.09, 231.11 |
| 24 | 249.148 | C15H20O3 | 248.1412 | 249.1485 | 105.07, 119.08, 128.06, 142.08, 143.09, 203.12, 213.13, 231.12 |
| 25 | 249.185 | C16H24O2 | 248.1776 | 249.1849 | 105.07, 119.08, 133.06, 147.11, 189.13, 217.12 |
| 26 | 249.221 | C17H28O | 248.2140 | 249.2213 | 105.07, 119.08, 133.10, 203.17 |
| 27 | 250.180 | C15H23NO2 | 249.1729 | 250.1802 | 105.07, 131.08, 145.10, 159.08, 187.15, 215.14 |
| 28 | 251.128 | C14H18O4 | 250.1205 | 251.1278 | 105.07, 107.08, 131.09, 145.10, 159.11, 187.11, 205.12, 215.11, 223.09, 233.11 |
| 29 | 251.164 | C15H22O3 | 250.1569 | 251.1642 | 105.07, 119.08, 131.08, 145.10, 147.11, 187.13, 215.12, 233.14 |
| 30 | 253.143 | C14H20O4 | 252.1362 | 253.1434 | 105.07, 119.08, 131.09, 133.10, 171.12, 189.12, 199.11, 206.09, 217.13, 235.13 |
| 31 | 253.180 | C15H24O3 | 252.1725 | 253.1798 | 105.07, 119.08, 131.08, 133.10, 159.11, 189.12, 207.11, 217.13, 235.14 |
| 32 | 257.189 | C18H24O | 256.1827 | 257.1900 | 105.07, 131.08, 145.10, 159.11, 183.11, 201.13 |
| 33 | 259.169 | C17H22O2 | 258.1620 | 259.1693 | 131.08, 145.10, 157.10, 159.11, 182.11, 215.14 |
| 34 | 261.148 | C16H20O3 | 260.1412 | 261.1485 | 105.07, 131.08, 145.09, 159.10, 173.10, 201.12, 215.13, 244.14 |
| 35 | 261.184 | C17H24O2 | 260.1776 | 261.1849 | 105.07, 131.08, 145.10, 201.15, 215.14, 243.16 |
| 36 | 261.220 | C18H28O | 260.2140 | 261.2213 | 131.08, 145.10, 159.11, 173.13, 217.16, 243.15 |
| 37 | 263.128 | C15H18O4 | 262.1205 | 263.1278 | 115.05, 128.06, 129.07, 141.07, 143.09, 156.09, 171.12, 199.11, 217.11, 227.11, 245.13 |
| 38 | 265.143 | C15H20O4 | 264.1362 | 265.1434 | 105.07, 131.08, 145.10, 159.11, 173.13, 219.13, 229.12, 247.12 |
| 39 | 267.159 | C15H22O4 | 266.1518 | 267.1591 | 105.07, 119.08, 131.08, 143.09, 157.10, 185.13, 213.13, 231.14, 249.15 |
| 40 | 275.164 | C17H22O3 | 274.1569 | 275.1642 | 105.07, 129.07, 131.09, 145.10, 155.09, 201.10, 215.13, 247.14, 257.15 |
| 41 | 275.200 | C18H26O2 | 274.1933 | 275.2006 | 105.07, 119.08, 159.10, 187.11, 201.12, 215.13, 229.12, 247.16 |
| 42 | 277.180 | C17H24O3 | 276.1725 | 277.1798 | 105.07, 119.08, 133.10, 147.12, 217.16, 231.17 |
| 43 | 285.125 | C15H24O5 | 284.1624 | 285.1697 | 105.07, 131.09, 143.09, 157.10, 185.13, 213.13, 221.11, 267.12 |
| 44 | 286.215 | C19H27NO | 285.2093 | 286.2165 | 131.07, 135.09, 145.09, 159.10, 164.11, 212.12, 233.15, 268.20 |
| 45 | 291.159 | C17H22O4 | 290.1518 | 291.1591 | 128.06, 131.09, 143.08, 157.10, 185.13, 213.13, 231.14 |
| 46 | 307.154 | C17H22O5 | 306.1467 | 307.1540 | 105.07, 131.09, 145.10, 159.11, 187.10, 205.09, 233.12, 261.15, 289.15 |
| 47 | 311.186 | C17H26O5 | 310.1780 | 311.1853 | 105.07, 131.08, 145.10, 159.10, 173.12, 201.12, 229.12, 247.13, 265.11, 283.15 |
| 48 | 321.242 | C20H32O3 | 320.2351 | 321.2424 | 105.07, 107.06, 119.08, 133.10, 159.12, 177.13, 201.15, 219.16, 229.16, 275.18 |
| 49 | 335.185 | C19H26O5 | 334.1780 | 335.1853 | 131.09, 145.10, 157.10, 159.10, 173.12, 201.13, 229.14, 247.13, 271.13, 299.17 |

**Figure S1** UV spectrum of compound **1**

**Figure S2** HRESIMS spectrum of compound **1**

**Figure S3** ^1^H NMR (500 MHz) spectrum of compound **1** in CDCl_3_

__

**Figure S4** ^13^C NMR (125 MHz) spectrum of compound **1** in CDCl_3_

__

**Figure S5** DEPT135 (125 MHz) spectrum of compound **1** in CDCl_3_

__

**Figure S6** ^1^H-^1^H COSY (500 MHz) spectrum of compound **1** in CDCl_3_

__

**Figure S7** HSQC (500 MHz) spectrum of compound **1** in CDCl_3_

__

**Figure S8** HMBC (500 MHz) spectrum of compound **1** in CDCl_3_

__

**Figure S9** NOESY (500 MHz) spectrum of compound **1** in CDCl_3_

**Figure S10** UV spectrum of compound **2**

**Figure S11** HRESIMS spectrum of compound **2**

**Figure S12** ^1^H NMR (500 MHz) spectrum of compound **2** in CDCl_3_

__

**Figure S13** ^13^C NMR (125 MHz) spectrum of compound **2** in CDCl_3_

__

**Figure S14** DEPT135 (125 MHz) spectrum of compound **2** in CDCl_3_

__

**Figure S15** ^1^H-^1^H COSY (500 MHz) spectrum of compound **2** in CDCl_3_

__

**Figure S16** HSQC (500 MHz) spectrum of compound **2** in CDCl_3_

__

**Figure S17** HMBC (500 MHz) spectrum of compound **2** in CDCl_3_

__

**Figure S18** NOESY (500 MHz) spectrum of compound **2** in CDCl_3_

**Figure S19** ^1^H NMR (500 MHz) spectrum of compound **2** in DMSO-*d*_6_

__

**Figure S20** ^13^C NMR (125 MHz) spectrum of compound **2** in DMSO-*d*_6_

**Figure S21** DEPT135 (125 MHz) spectrum of compound **2** in DMSO-*d*_6_

__

**Figure S22** ^1^H-^1^H COSY (500 MHz) spectrum of compound **2** in DMSO-*d*_6_

__

**Figure S23** HSQC (500 MHz) spectrum of compound **2** in DMSO-*d*_6_

__

**Figure S24** HMBC (500 MHz) spectrum of compound **2** in DMSO-*d*_6_

__

**Figure S25** ROESY (500 MHz) spectrum of compound **2** in DMSO-*d*_6_

**Figure S26** UV spectrum of compound **6**

**Figure S27** HRESIMS spectrum of compound **6**

**Figure S28** ^1^H NMR (500 MHz) spectrum of compound **6** in CDCl_3_

__

**Figure S29** ^13^C NMR (125 MHz) spectrum of compound **6** in CDCl_3_

__

**Figure S30** DEPT135 (125 MHz) spectrum of compound **6** in CDCl_3_

__

**Figure S31** ^1^H-^1^H COSY (500 MHz) spectrum of compound **6** in CDCl_3_

__

**Figure S32** HSQC (500 MHz) spectrum of compound **6** in CDCl_3_

__

**Figure S33** HMBC (500 MHz) spectrum of compound **6** in CDCl_3_

__

**Figure S34** NOESY (500 MHz) spectrum of compound **6** in CDCl_3_

**Figure S35** UV spectrum of compound **7**


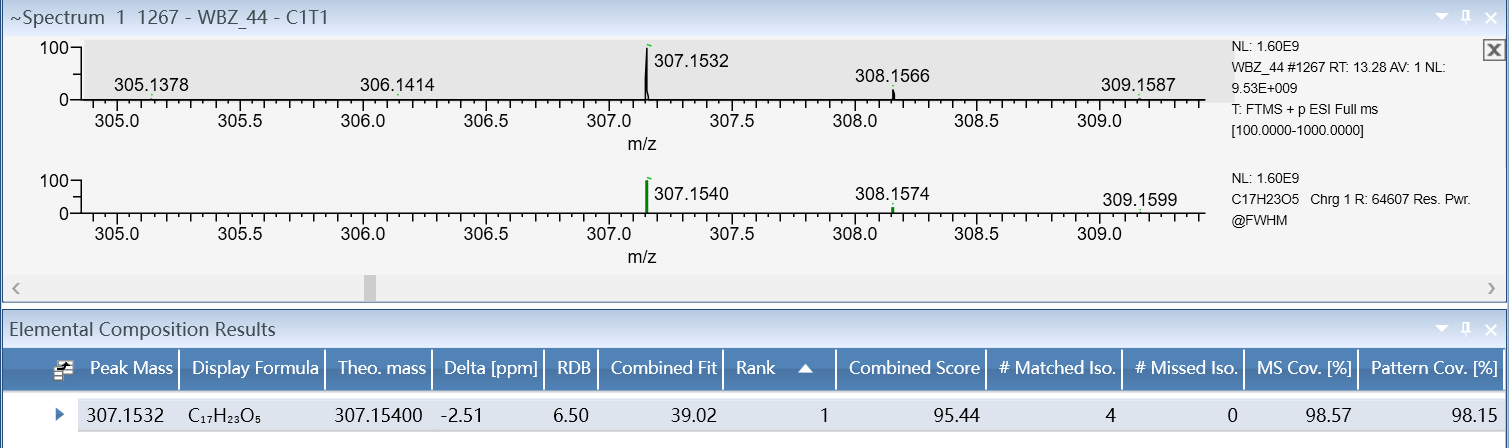


**Figure S36** HRESIMS spectrum of compound **7**

**Figure S37** ^1^H NMR (500 MHz) spectrum of compound **7** in CDCl_3_

__

**Figure S38** ^13^C NMR (125 MHz) spectrum of compound **7** in CDCl_3_

**Figure S39** DEPT135 (125 MHz) spectrum of compound **7** in CDCl_3_

__

**Figure S40** ^1^H-^1^H COSY (500 MHz) spectrum of compound **7** in CDCl_3_

__

**Figure S41** HSQC (500 MHz) spectrum of compound **7** in CDCl_3_

__

**Figure S42** HMBC (500 MHz) spectrum of compound **7** in CDCl_3_

__

**Figure S43** NOESY (500 MHz) spectrum of compound **7** in CDCl_3_

**Figure S44** ^1^H NMR (500 MHz) spectrum of compound **7** in DMSO-*d*_6_

**Figure S45** ROESY (500 MHz) spectrum of compound **7** in DMSO-*d*_6_
